# Effect of *Sargassum aquifolium* Juice on Lipid Profile and Inflammatory Cytokines in Hypercholesterolemic Rats

**DOI:** 10.1155/ijfo/3668883

**Published:** 2025-07-01

**Authors:** Muhamad Firdaus, Retno Tri Astuti, Yoga Dwi Jatmiko, Herlina Pratiwi

**Affiliations:** ^1^Faculty of Fisheries and Marine Science, Universitas Brawijaya, Malang, Indonesia; ^2^Department of Biology, Faculty of Mathematics and Natural Sciences, Universitas Brawijaya, Malang, Indonesia; ^3^Faculty of Veterinary Medicine, Universitas Brawijaya, Malang, Indonesia

**Keywords:** hypercholesterolemic, inflammatory cytokines, lipid profile, niacin, *Sargassum aquifolium* juice

## Abstract

Obesity, a global health burden, is marked by increased lipid levels and chronic inflammation. *Sargassum aquifolium* is a brown seaweed rich in bioactive compounds that may improve lipid profile and inflammation. This study is aimed at evaluating the potential of *S. aquifolium* juice in modulating lipid profiles and inflammatory responses in hypercholesterolemic rats. High-performance liquid chromatography–high-resolution mass spectrometry (HPLC–HRMS) analysis identified bioactive compounds, including niacin, whose potential antihypercholesterolemic effect was supported by in silico PASS prediction. Male Wistar rats were divided into five groups: normal, hypercholesterolemic, and hypercholesterolemic rats fed *S. aquifolium* juice once, twice, and thrice daily for 120 days. The results showed that administration of *S. aquifolium* juice, especially twice daily, significantly reduced TG and total cholesterol, raised HDL, lowered AI and malondialdehyde (MDA), and modulated IL-4, TNF-*α*, and IL-1*β* expression by CD4^+^ cells. In hypercholesterolemic rats, IL-4 expression was lower, and TNF-*α* and IL-1*β* were higher than normal. However, *S. aquifolium* juice increased IL-4 and decreased TNF-*α* and IL-1*β*, suggesting an immunomodulatory effect. Niacin in *S. aquifolium* juice may improve lipid profiles and suppress inflammatory cytokines via NF-*κ*B inhibition and NLRP3 inflammasome modulation. Overall, these findings highlight the potential of *S. aquifolium* juice as a natural antihypercholesterolemic agent for managing hypercholesterolemia and preventing atherosclerosis, though further larger scale investigations are needed.

## 1. Introduction

*Obesity* is a complex condition characterized by excessive accumulation of body fat [1]. The buildup of fat can harm health and has become a serious global issue. According to updated data from the World Health Organization (WHO), in 2022, more than 2.5 billion adults were overweight, and over 890 million were classified as obese. This prevalence indicates that about 43% of the world's adult population—43% of men and 44% of women—are obese, with obesity rates highest in the Americas (67%) and lowest in Southeast Asia and Africa (31%) [2]. Obesity can lead to various health consequences, both acute and chronic [3]. Acutely, it may increase the risk of postoperative complications and musculoskeletal problems. In contrast, chronic obesity contributes to an elevated risk of diseases such as cardiovascular disease, Type 2 diabetes, and cancers like breast, prostate, and colon [4–6].

During acute obesity, there is an increase in free fatty acids and proinflammatory cytokines (TNF-*α* amd IL-1*β*); these markers remain persistently elevated in chronic obesity [7, 8]. Such inflammation is closely related to disrupted lipid profiles, wherein excess free fatty acids and triglycerides (TGs) coincide with lower high-density lipoprotein (HDL) cholesterol (Chol). Consequently, surplus fat accumulation can cause macrophage infiltration, increase proinflammatory cytokines (TNF-*α* and IL-1*β*), and reduce anti-inflammatory cytokines. In hypercholesterolemia, elevated low-density lipoprotein (LDL) in the blood oxidizes into oxidized LDL (oxLDL), which immune cells capture through scavenger receptors [9, 10]. This process activates CD4^+^ T cells that release TNF-*α* and IL-1*β*, creating a proinflammatory environment conducive to atherosclerotic plaque formation [11, 12].

Some natural antihypercholesterol ingredients improve lipid profiles by inhibiting Chol synthesis, promoting excretion, and modulating lipid metabolism. Compounds such as flavonoids and polyphenols have antioxidant properties, helping to avert LDL oxidation—a critical step in atherosclerosis [13]. Additionally, these agents may exhibit immunomodulatory effects by suppressing proinflammatory cytokines (TNF-*α* and IL-1*β*) and boosting anti-inflammatory cytokines, thus mitigating chronic inflammation [13]. Vitamins and bioactive molecules, including niacin and polyphenols, have been reported to reduce proinflammatory cytokine production partly by inhibiting the nucleotide-binding oligomerization domain, leucine-rich repeat-containing gene family, pyrin domain-containing 3 (NLRP3) inflammasome [14–16].

Brown seaweeds, particularly in the genus *Sargassum*, have garnered interest as potential sources of antihypercholesterol compounds. These seaweeds are rich in bioactive substances such as fucoidan, phlorotannins, and carotenoids, which have been observed to improve lipid profiles and modulate proinflammatory mediators [17–20]. However, molecular or histological analyses confirming the specific mechanisms of these bioactives are still limited.

*Sargassum aquifolium* is abundant in tropical and subtropical waters and has traditionally been employed to address conditions such as hypertension and atherosclerosis [20]. Prior findings show that constituents (e.g., fucoidan and fucosterol) reduce proinflammatory cytokines [21–28], potentially aiding in obesity-related inflammation [29–31]. Preparing *S. aquifolium* as juice can offer a more straightforward dietary approach for delivering bioactive compounds, and the rats in the present study were maintained on an atherogenic diet under standardized environmental conditions to ensure uniformity [32–36].

Notwithstanding these prior studies, a clear research gap persists regarding the specific contribution of *Sargassum*-derived bioactives in modulating lipid metabolism and immune responses in hypercholesterolemic models. Therefore, the present study is aimed at evaluating whether *S. aquifolium* juice can enhance lipid profiles and modulate key inflammatory cytokines (IL-4, TNF-*α*, and IL-1*β*) in hypercholesterolemic rats. By focusing on these parameters, we seek to fill the research void in understanding how *S. aquifolium*'s bioactive components influence lipid metabolism and immune function, potentially offering a novel dietary strategy for managing hypercholesterolemia.

## 2. Materials and Methods

### 2.1. Material

The sample was obtained from Ekas Bay, West Nusa Tenggara, Indonesia. It was morphologically identified at the National Research and Innovation Agency, Jakarta, Indonesia (Specimen Code 1346-23057-1) and named *S. aquifolium*. All visible debris was removed, and the seaweed was rinsed with distilled water before juicing. To maintain sample consistency, the samples were juiced using a slow juicer (Philips Model HR1889).

### 2.2. High-Performance Liquid Chromatography–High-Resolution Mass Spectrometer (HPLC–HRMS) Analysis

Bioactive identity determination of *S. aquifolium* juice was performed using a HPLC–HRMS (Thermo Scientific Dionex Ultimate 3000 RSLCnano). Following Firdaus et al. [18], the juice was filtered and injected, and the resulting peaks were analyzed to identify bioactive components. PASS software (in silico) served as a preliminary screening tool; hence, molecular experiments are recommended to validate identified compounds further.

### 2.3. In Silico Analysis

Antihypercholesterolemic activity prediction of the bioactive compounds identified in *S. aquifolium* juice was conducted in silico using the canonical SMILES PASS (substance spectrum prediction) online software (http://pharmaexpert.ru/passonline/org). The probability of activity (Pa) value was used as a benchmark, with Pa > 0.7 indicating potential antihypercholesterolemic effects.

### 2.4. Experimental Animals

Male Wistar rats (*Rattus norvegicus*) aged 2.5–3 months (body weight 130–150 g) were used in this study. The rats were housed under uniform conditions at 25°C ± 2°C, under a 12 h light/12 h dark cycle. They were fed a standardized diet ad libitum during acclimatization and had free access to distilled water. Each group included five rats, as approved by the Ethics Committee of Brawijaya University (113-KEP-UB-2023).

### 2.5. Experimental Design

Five groups were established: (A) normal, (B) hypercholesterolemic, (C) hypercholesterolemic + *S. aquifolium* juice once daily, (D) hypercholesterolemic + juice twice daily, and (E) hypercholesterolemic + juice thrice daily. All groups were maintained in the same environmental conditions. The atherogenic diet for hypercholesterolemic groups (B, C, D, and E) comprised 10% saturated fats and 2% Chol plus 0.2% cholic acid, while the normal group (A) received a standard laboratory diet [37] (Table 1). Groups A and B were administered 1 mL of distilled water orally. Groups C, D, and E received 1 mL of *S. aquifolium* juice after the principal meal to ensure consistent nutritional intake. Rats were observed for 120 days following a 7-day acclimatization period.

### 2.6. Lipid Profile Analysis

Serum samples were collected from the orbital sinus and centrifuged at 5000 rpm for 10 min. A 500 *μ*L portion of serum was reacted with 1000 *μ*L of reagent (ABX Pentra Triglycerides CP, ABX Pentra Cholesterol CP, and ABX Pentra HDL Direct CP), and levels of TG, total Chol, and HDL were measured using a Pentra C200 analyzer (Horiba).

### 2.7. Atherogenic Index (AI)

The AI was calculated from the ratio of TG to HDL, following Fernández-Macías et al. [38]:

 $$\begin{array}{c} \text{AI}=\text{Log}\left(\frac{\text{TG}}{\text{HDL}}\right). \end{array}$$

Higher AI values indicate a greater atherosclerosis risk.

### 2.8. Malondialdehyde (MDA) Analysis

MDA was measured using the thiobarbituric acid (TBA) method [39]. Blood samples were centrifuged at 6000 rpm for 15 min at 4°C. A 1 mL portion of the supernatant was stored at −20°C in the dark until analysis. For quantification, 250 *μ*L of the thawed sample was mixed with 625 *μ*L of 40% trichloroacetic acid, 50 *μ*L sodium thiosulfate, 100 *μ*L 1 N HCl, and 2 mL distilled water. The solution was vortexed, heated at 100°C for 15 min, sealed, and then centrifuged at 4000 rpm for 10 min. The supernatant was diluted to 3 mL with distilled water, and absorbance was read at 532 nm using a spectrophotometer.

### 2.9. Flow Cytometry Analysis

Peripheral blood mononuclear cells (PBMCs) were isolated to measure the percentage of IL-4, IL-1*β*, and TNF-*α*. Flow cytometry was conducted using BD FACSMelody and BD FACSChorus software. Staining involved FITC-CD4^+^ antirat (BioLegend, 201505), AF647-IL-4 antirat (Santa Cruz Biotechnology, sc-53084), AF647-IL-1*β* antirat (Santa Cruz Biotechnology, sc-515598), and AF647-TNF-*α* antirat (Bios, bs-2081R). The procedure followed the manufacturer's instructions, and the data were expressed as the percentage of cytokine-positive cells among CD4^+^ T lymphocytes.

### 2.10. Data Analysis

Statistical analysis was performed via one-way ANOVA, followed by the least significant difference (LSD) post hoc test at *p* < 0.05. Results are presented as mean ± standard deviation (SD). IBM SPSS Statistics Version 16.0 was used for all data processing.

## 3. Results and Discussion

### 3.1. HPLC–HRMS and In Silico Analysis

The results of untargeted HPLC–HRMS bioactive identity analysis on *S. aquifolium* juice found 18 compounds, including betaine, DL-carnitine, choline, nicotinic acid (niacin), caprolactam, triethyl phosphate, DEET, 2,2,6,6-tetramethyl-1-piperidinol, D-(+)-camphor, 1-tetradecylamine, 3,5-di-tert-butyl-4-hydroxybenzaldehyde, and tetranor-12(S)-HETE. Previous studies indicate that other *Sargassum* species contain numerous bioactive substances, such as *Sargassum oligocystum*, *Sargassum cristaefolium*, and *Sargassum echinocarpum* [18, 40]. In this study, in silico PASS software (Pa = 0.883) identified niacin as a key candidate for potential Chol-lowering activity, although molecular assays are needed to confirm its specific function fully. Niacin can help lower Chol and TG levels by modulating their synthesis and slowing HDL catabolism [41]. Nevertheless, caution is advised against overreliance on computational predictions; further in vivo corroboration would strengthen these preliminary findings.

### 3.2. *S. aquifolium* Juice Effects on Lipid Profiles

Table 2 shows the TG, total Chol, HDL, AI, and MDA levels in normal, hypercholesterolemic, and hypercholesterolemic rats treated with *S. aquifolium* juice once, twice, and three times daily.

Administration of *S. aquifolium* juice significantly improved the lipid profile of hypercholesterolemic rats, with the twice-daily treatment (SaJ2) yielding the most pronounced effect. This finding aligns with previous studies reporting that various *Sargassum* species can lower TGs and total Chol while increasing HDL levels in animal models [42–44]. These effects are likely attributable to the presence of fucoxanthin, dietary fiber, and niacin, which are known to reduce fatty acid synthesis, promote fatty acid oxidation, and inhibit key enzymes involved in Chol metabolism [41, 45].

The hypercholesterolemic control group exhibited elevated lipid parameters compared to both the normal and juice-treated groups (Table 2). Among the treated groups, those receiving the juice twice daily demonstrated the most significant improvement in lipid parameters. Similar results have been observed in studies using *Sargassum polycystum* and other brown seaweeds, showing lipid-lowering effects when incorporated into high-fat diets [44]. The active components in seaweed, such as fucoxanthin, can inhibit TG synthesis in adipose tissue and the liver, enhance faecal lipid excretion, and increase HDL levels. Dietary fibers also contribute to hypolipidemic effects by binding bile acids and reducing Chol absorption in the small intestine [45].

In addition to fucoxanthin and fiber, the presence of niacin in *S. aquifolium* juice may further explain its antihyperlipidemic properties. Niacin has been shown to inhibit TG synthesis and reduce HDL catabolism. Moreover, it modulates inflammatory responses by enhancing PUMA-G expression in macrophages, thereby reducing fatty acid availability for TG formation. It also increases peroxisome proliferator-activated receptor gamma (PPAR*γ*) expression, preventing HDL degradation [41].

While more frequent administration generally enhances bioavailability, the twice-daily treatment proved more effective than once- or thrice-daily dosing. This is consistent with reports suggesting that excessive intake of bioactive-rich juices may lead to antagonistic interactions or reduced efficacy due to loss of synergistic effects or potential toxicity [35, 36, 46–48].

The AI was significantly higher in hypercholesterolemic controls than in normal rats and juice-treated groups (Table 2). Treatment with *S. aquifolium* juice effectively reduced AI, indicating a lower cardiovascular risk. This is consistent with findings on other *Sargassum* species (*Sargassum pallidum*, *Sargassum horneri*, *Sargassum fusiforme*, *Sargassum thunbergii*, and *S. polycystum*), which have demonstrated antiatherogenic effects in vivo [49–51]. The reduction in AI in this study may also be linked to niacin's role in inhibiting TG synthesis and stabilizing HDL levels [52].

Furthermore, MDA levels—a biomarker of lipid peroxidation and oxidative stress—were significantly lower in the juice-treated groups than in untreated hypercholesterolemic rats (Table 1). This suggests that *S. aquifolium* juice offers protection against oxidative damage. Prior studies have reported that *S. polycystum* extracts reduce MDA levels in diabetic models [43]. Niacin may contribute to this effect by inhibiting hepatic LDL secretion, decreasing its susceptibility to oxidation [41].

### 3.3. *S. aquifolium* Juice Effects on IL-4 Expression

**Figure 1:**
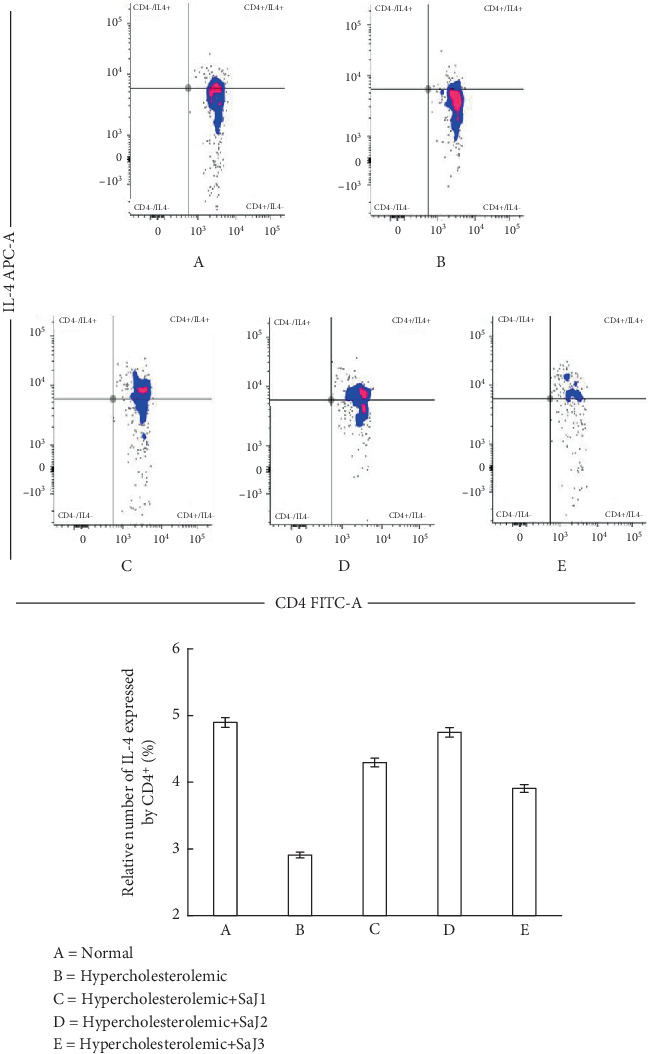
The relative percentage of IL-4 expression by CD4^+^ in normal and hypercholesterolemic rats affected by *S. aquifolium* juice.

The relative percentage of IL-4 expression by CD4^+^ in experimental animals showed differences (*p* < 0.05).  Figure 1 shows the expression of IL-4 in normal, hypercholesterolemic, and hypercholesterolemic rats treated with *S. aquifolium* juice.

Figure 1 and Table 3 illustrate the relative percentage of IL-4 expression by CD4^+^ cells. IL-4 expression was highest in normal rats and lowest in hypercholesterolemic rats. CD4^+^ T helper cells, particularly the Th2 subtype, secrete IL-4, critical in humoral immunity and anti-inflammatory responses. Under normal physiological conditions, immune homeostasis is maintained through a balance of pro- and anti-inflammatory cytokines. This balance is disrupted in hypercholesterolemic conditions, resulting in heightened proinflammatory activity and diminished IL-4 expression, possibly contributing to disease progression such as atherosclerosis [53–55].

In this study, administration of *S. aquifolium* juice increased IL-4 expression in hypercholesterolemic rats, suggesting restoration of anti-inflammatory activity. Among the treated groups, twice-daily administration (SaJ2) produced IL-4 levels most comparable to those in normal rats, indicating enhanced immunomodulatory effects. This may reflect the optimal exposure to bioactive compounds in the juice, as once-daily dosing may not sustain sufficient levels. At the same time, thrice-daily administration could lead to saturation or adverse interactions among the active constituents [56–60].

Brown seaweeds, including *Sargassum* species, contain polysaccharides such as fucoidan, which have been shown to promote Th2 responses and increase IL-4 expression in hypercholesterolemic models [56–62]. In line with this, the present findings indicate that *S. aquifolium* juice may exert its immunomodulatory effects partly through such bioactives. Notably, the group receiving the juice three times daily exhibited lower IL-4 expression than the twice-daily group, highlighting a potential threshold beyond which additional exposure may not yield added benefits and could even impair immune balance.

One compound of particular interest in *S. aquifolium* is niacin, which was identified via HPLC–HRMS analysis and in silico prediction. Niacin modulates immune responses by binding to the GPR109A receptor on immune cells such as macrophages and dendritic cells. This interaction inhibits proinflammatory signaling pathways like NF-*κ*B and MAPK while promoting the expression of anti-inflammatory cytokines such as IL-10 and TGF-*β* [63, 64]. Although direct in vivo confirmation in the context of *S. aquifolium* juice is lacking, the observed upregulation of IL-4 may reflect the compound's systemic immunomodulatory influence.

In addition, in vitro and in vivo studies have shown that niacin suppresses the production of proinflammatory cytokines (e.g., TNF-*α* and IL-1*β*) and promotes Th2 polarization by enhancing regulatory and anti-inflammatory responses [63]. This may help counteract the proinflammatory state associated with hypercholesterolemia and improve immune equilibrium.

### 3.4. *S. aquifolium* Juice Effects on TNF-*α* Expression

**Figure 2:**
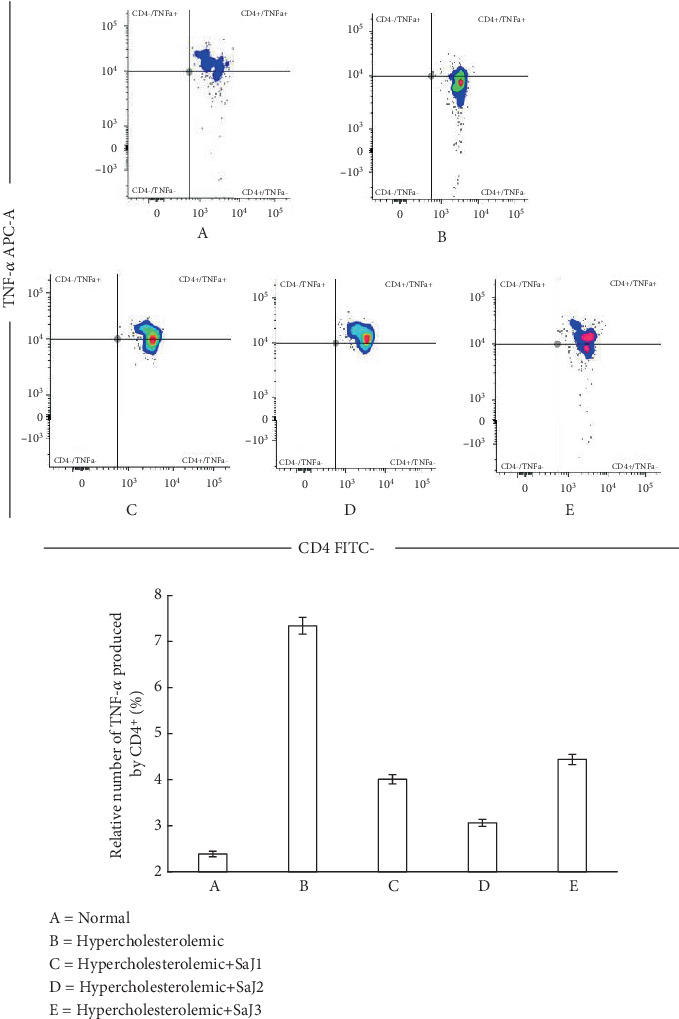
The relative percentage of TNF-*α* expression by CD4^+^ in normal and hypercholesterolemic rats affected by *S. aquifolium* juice.

TNF-*α* expression in experimental animals showed differences (*p* < 0.05).  Figure 2 shows the expression of TNF-*α* in normal, hypercholesterolemic, and hypercholesterolemic rats treated with *S. aquifolium* juice.

Figure 2 and Table 3 present the relative percentage of TNF-*α* expression by CD4^+^ cells in normal and hypercholesterolemic rats, with or without *S. aquifolium* juice treatment. The hypercholesterolemic control group exhibited the highest TNF-*α* expression, consistent with elevated inflammation associated with increased levels of LDL and oxidized LDL [65–67]. Hypercholesterolemia alters immune regulation, where activated CD4^+^ T cells, particularly Th1, interact with macrophages and endothelial cells, leading to the upregulation of proinflammatory cytokines, including TNF-*α* [65–69].

In the present study, administration of *S. aquifolium* juice influenced TNF-*α* expression by CD4^+^ cells. Among the treatment groups, the twice-daily regimen (SaJ2) resulted in the most substantial reduction in TNF-*α* levels, suggesting an optimal exposure to anti-inflammatory compounds. Interestingly, the group receiving juice three times daily exhibited higher TNF-*α* expression than the twice-daily group, which may reflect diminished returns or potential antagonistic effects from excessive exposure.

Several bioactive compounds in brown seaweeds, such as fucoidan and phlorotannins, have demonstrated the capacity to reduce TNF-*α* expression. For example, phlorotannins from *Ecklonia cava* suppressed TNF-*α* production and mRNA expression in macrophage cells [70]. At the same time, oral fucoidan from *S. horneri* significantly reduced TNF-*α* in colitis-induced rats and macrophage models via NF-*κ*B inhibition [71, 72]. Similar anti-inflammatory effects were reported with topical *Sargassum serratifolium* extract [73]. These compounds exert their effects through multiple signaling pathways, including inhibition of NF-*κ*B and AP-1 transcription factors, as well as downregulation of MAPK pathways (ERK, JNK, and p38), which are involved in the transcriptional regulation of TNF-*α* [74–76].

The frequency of juice administration likely affects these compounds' exposure duration and bioavailability. Once-daily administration led to a modest decrease in TNF-*α*expression, while twice-daily dosing allowed for sustained interaction with immune cells, leading to a more consistent anti-inflammatory effect. In contrast, thrice-daily administration may surpass the threshold for optimal benefit, potentially leading to desensitization or compensatory proinflammatory signaling [77, 78].

Among the identified bioactives, niacin, detected in *S. aquifolium* juice via HPLC–HRMS and predicted to possess immunomodulatory potential, has been extensively studied for its role in lowering TNF-*α*. Experimental and clinical studies indicate that niacin reduces TNF-*α* production through GPR109A receptor activation, inhibiting NF-*κ*B via the cAMP–PKA pathway [79–86]. However, given that this study did not isolate niacin's effects or perform receptor-specific assays, these mechanisms remain speculative in this context. The observed reduction in TNF-*α* may thus reflect a synergistic interaction between multiple compounds rather than the action of a single agent.

### 3.5. *S. aquifolium* Juice Effects on IL-1*β* Expression

**Figure 3:**
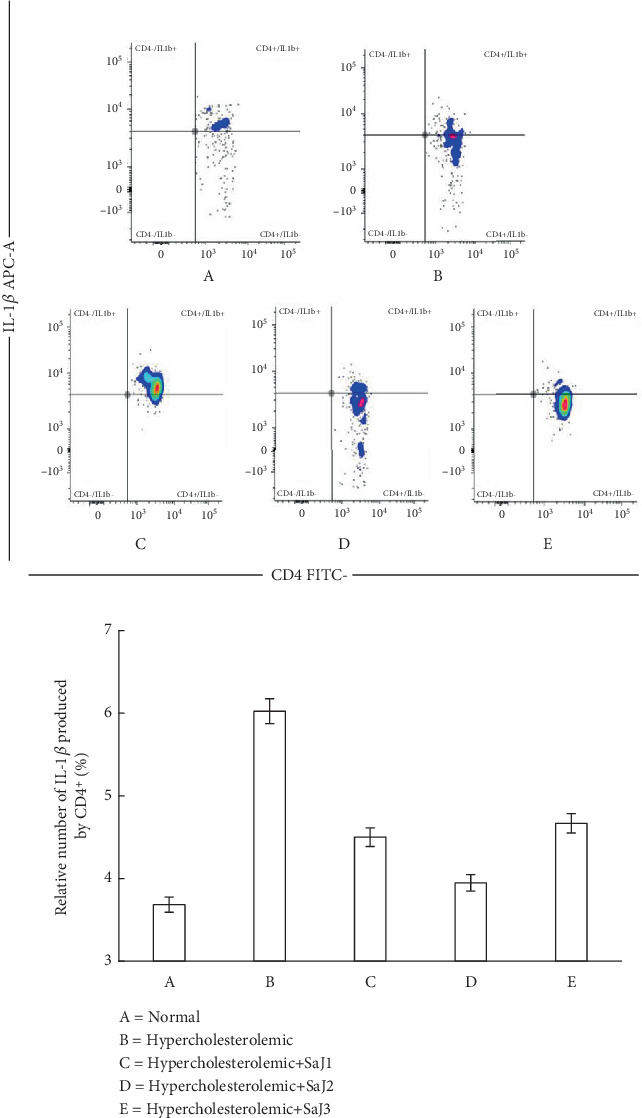
The relative percentage of IL-1*β* expression by CD4^+^ in normal and hypercholesterolemic rats affected by *S. aquifolium* juice.

IL-1*β* expression in experimental animals showed differences (*p* < 0.05).  Figure 3 shows the expression of IL-1*β* in normal, hypercholesterolemic, and hypercholesterolemic rats treated with *S. aquifolium* juice.

Figure 3 and Table 3 show the relative percentage of IL-1*β* expression by CD4^+^ cells across treatment groups. Hypercholesterolemic rats without treatment exhibited significantly elevated IL-1*β* levels compared to normal rats, indicating an enhanced inflammatory response. Administration of *S. aquifolium* juice reduced IL-1*β* expression, with the most notable reduction observed in the group receiving the juice twice daily.

IL-1*β* is a key proinflammatory cytokine regulated by the NLRP3 inflammasome and the NF-*κ*B signaling pathway. In hypercholesterolemic conditions, elevated levels of oxLDL can activate these pathways in CD4^+^ cells, resulting in increased transcription and secretion of IL-1*β* [87, 88]. Excess Chol also promotes NLRP3 inflammasome activation, which triggers caspase-1–mediated maturation of IL-1*β*. Studies have shown that CD4^+^ cells from hyperlipidemic models exhibit upregulation of NLRP3 components and increased IL-1*β* production, particularly in adipose and splenic tissues [88].

The present study suggests that bioactive compounds in *S. aquifolium* may modulate these inflammatory pathways. Brown seaweeds contain fucoxanthin, phlorotannins, and sulfated polysaccharides, which have been reported to inhibit IL-1*β* expression. These compounds suppress the activation of NF-*κ*B through various mechanisms, including inhibition of I*κ*B phosphorylation and nuclear translocation, as well as direct interference with transcription factor binding to IL-1*β* promoter regions [89–92]. Additionally, they may downregulate MAPK signaling pathways such as p38 and JNK, which are also involved in IL-1*β* gene expression.

Furthermore, seaweed-derived bioactives may attenuate inflammasome activation by reducing oxidative stress, improving mitochondrial function, and dampening cellular danger signals. This multifaceted mechanism likely contributes to decreased IL-1*β* expression in treated animals. In this study, once-daily juice administration produced moderate effects, while thrice-daily dosing did not enhance the response further and, in some cases, showed higher IL-1*β* levels than twice-daily administration, potentially due to bioactive overload and loss of efficacy.

Niacin, identified in *S. aquifolium* juice via HPLC–HRMS and in silico prediction, may also contribute to the observed anti-inflammatory effects. Although its mechanisms are well-documented, such as GPR109A-mediated suppression of NF-*κ*B and inhibition of NLRP3 inflammasome activation, this study did not directly isolate or confirm its role. Therefore, the involvement of niacin remains speculative and should be interpreted as part of a synergistic effect rather than a sole contributor [93, 94].

Collectively, the findings of this study indicate that *S. aquifolium* juice improves lipid profiles, marked by reductions in TGs and total Chol, and increases in HDL, while also attenuating inflammation through the modulation of key cytokines, including TNF-*α*, IL-1*β*, and IL-4. These effects were most consistently observed with twice-daily administration, suggesting that this dosing frequency may offer optimal therapeutic benefits.

However, the small sample size (*n* = 5 per group) limits the study's statistical power, potentially affecting the robustness and reproducibility of the results. This limitation also constrains the generalizability of the findings to broader populations. Although the trend across multiple parameters supports the benefit of twice-daily dosing, the differences across treatment frequencies should be interpreted cautiously due to limited group size and potential intragroup variability.

Identifying niacin via HPLC–HRMS profiling and PASS analysis suggests a possible role in the observed anti-inflammatory effects, particularly through its known interactions with GPR109A and its regulatory impact on NF-*κ*B and NLRP3 inflammasome pathways. Nonetheless, these mechanistic insights remain speculative without direct validation through molecular or histological assays. Other compounds commonly present in brown seaweed—such as fucoxanthin, fucoidan, and phlorotannins—are likely to act synergistically and contribute meaningfully to the observed outcomes.

This study provides preliminary but promising evidence supporting the antihypercholesterolemic and immunomodulatory potential of *S. aquifolium* juice. Future studies with larger cohorts and more rigorous molecular analyses, including niacin-only control groups and pathway-specific assays, are needed to confirm the mechanisms and strengthen the translational relevance of these findings.

## 4. Conclusion

*S. aquifolium* juice contains niacin and other bioactive compounds identified by HPLC–HRMS, showing the potential to enhance lipid profiles and modulate inflammatory cytokines in hypercholesterolemic rats. Despite using only five rats per group, the present study indicates that twice-daily administration of *S. aquifolium* juice yields the most pronounced improvements in TGs, total Chol, HDL levels, AI, and MDA. Furthermore, modulating IL-4, TNF-*α*, and IL-1*β* expression suggests an immunomodulatory effect that could reduce atherosclerosis risk. Nevertheless, caution is warranted regarding broader applicability, and future research should include larger sample sizes, additional positive controls, and molecular validations of niacin's mechanism. These findings support the potential use of *S. aquifolium* juice as a natural antihypercholesterolemic agent and highlight the need for further comprehensive studies to confirm its therapeutic relevance.

## Figures and Tables

**Table 1:** Composition of regular and atherogenic diet.

**Composition**	**Regular**	**Atherogenic**
Protein (%)	13.93 ± 0.22^a^	13.33 ± 0.38^a^
Lipid (%)	4.02 ± 0.12^a^	14.63 ± 0.51^b^
Mineral (%)	2.71 ± 0.29^a^	2.41 ± 0.33^a^
Vitamin (%)	0.50 ± 0.02^a^	0.49 ± 0.02^a^
Fiber (%)	1.71 ± 0.08^b^	1.32 ± 0.13^a^
Carbohydrate (%)	70.25 ± 0.65^b^	62.40 ± 0.83^a^
Water (%)	5.98 ± 0.06^a^	5.85 ± 0.18^a^
Energy (kcal/kg)	3822.56 ± 51.5^a^	4267.6 ± 107.8^b^

*Note:* Values are expressed as mean ± SD; different superscript letters indicate significant differences at *p* < 0.05.

**Table 2:** *S. aquifolium* juice affects lipid profiles, AI, and MDA levels in hypercholesterolemic rats.

**Treatments**	**TG (mg/dL)**	**Chol (mg/dL)**	**HDL (mg/dL)**	**AI**	**MDA (ng/mL)**
Normal	42 ± 2.7^a^	37 ± 3.5^a^	40 ± 3.3^d^	0.02	0.108 ± 0.008^a^
Hypercholesterolemic	150 ± 7.8^e^	98 ± 4.6^e^	12 ± 1.1^a^	1.09	0.236 ± 0.032^d^
Hypercholesterolemic + SaJ1	85 ± 4.9^c^	56 ± 3.4^c^	20 ± 1.7^b^	0.63	0.156 ± 0.023^c^
Hypercholesterolemic + SaJ2	58 ± 3.2^b^	48 ± 2.4^b^	28 ± 1.9^c^	0.32	0.132 ± 0.018^b^
Hypercholesterolemic + SaJ3	97 ± 3.4^d^	86 ± 2.6^d^	18 ± 1.5^b^	0.73	0.178 ± 0.016^c^

*Note:* Values are expressed as mean ± SD; different superscript letters indicate significant differences at *p* < 0.05.

**Table 3:** *S. aquifolium* juice affects the relative percentage of IL-4, TNF-*α*, and IL-1*β* expression by CD4^+^ in normal and hypercholesterolemic rats.

**Treatments**	**IL-4 (mg/dL)**	**TNF-*α* (mg/dL)**	**IL-1*β* (mg/dL)**
Normal	4.89 ± 0.014^e^	2.39 ± 0.013^a^	3.68 ± 0.013^a^
Hypercholesterolemic	2.91 ± 0.025^a^	7.34 ± 0.052^e^	6.02 ± 0.013^e^
Hypercholesterolemic + SaJ1	4.29 ± 0.085^c^	4.01 ± 0.028^c^	4.49 ± 0.030^c^
Hypercholesterolemic + SaJ2	4.75 ± 0.021^d^	3.06 ± 0.059^b^	3.95 ± 0.033^b^
Hypercholesterolemic + SaJ3	3.91 ± 0.019^b^	4.44 ± 0.015^d^	4.67 ± 0.021^d^

*Note:* Values are expressed as mean ± SD; different superscript letters indicate significant differences at *p* < 0.05.

## Data Availability

The data that support the findings of this study are available from the corresponding author upon reasonable request.

## References

[1] Nuttall F. Q. (2015). Body Mass Index: Obesity, BMI, and Health: A Critical Review. *Nutrition Today*.

[2] World Health Organization (2024). Obesity and Overweight. https://www.who.int/news-room/fact-sheets/detail/obesity-and-overweight.

[3] Blüher M. (2019). Obesity: Global Epidemiology and Pathogenesis. *Nature Reviews Endocrinology*.

[4] Abdelaal M., le Roux C. W., Docherty N. G. (2017). Morbidity and Mortality Associated With Obesity. *Annals of Translational Medicine*.

[5] Guh D. P., Zhang W., Bansback N., Amarsi Z., Birmingham C. L., Anis A. H. (2009). The Incidence of Co-Morbidities Related to Obesity and Overweight: A Systematic Review and Meta-Analysis. *BMC Public Health*.

[6] Manna P., Jain S. K. (2015). Obesity, Oxidative Stress, Adipose Tissue Dysfunction, and the Associated Health Risks: Causes and Therapeutic Strategies. *Metabolic Syndrome and Related Disorders*.

[7] Izaola O., de Luis D., Sajoux I., Domingo J. C., Vidal M. (2015). Inflammation and Obesity (Lipoinflammation). *Nutricion Hospitalaria*.

[8] Ellulu M. S., Patimah I., Khaza'ai H., Rahmat A., Abed Y. (2017). Obesity and Inflammation: The Linking Mechanism and the Complications. *Archives of Medical Science*.

[9] Tedgui A., Mallat Z. (2006). Cytokines in Atherosclerosis: Pathogenic and Regulatory Pathways. *Physiological Reviews*.

[10] Hansson G. K., Hermansson A. (2011). The Immune System in Atherosclerosis. *Nature Immunology*.

[11] Moss J. W. E., Ramji D. P. (2016). Cytokines: Roles in Atherosclerosis Disease Progression and Potential Therapeutic Targets. *Future Medicinal Chemistry*.

[12] Tousoulis D., Oikonomou E., Economou E. K., Crea F., Kaski J. C. (2016). Inflammatory Cytokines in Atherosclerosis: Current Therapeutic Approaches. *European Heart Journal*.

[13] Orekhov A. N., Sobenin I. A., Korneev N. V. (2013). Anti-Atherosclerotic Therapy Based on Botanicals. *Recent Patents on Cardiovascular Drug Discovery*.

[14] Zhang Y. J., Gan R. Y., Li S. (2015). Antioxidant Phytochemicals for the Prevention and Treatment of Chronic Diseases. *Molecules*.

[15] Lipszyc P. S., Cremaschi G. A., Zubilete M. Z. (2013). Niacin Modulates Pro-Inflammatory Cytokine Secretion. A Potential Mechanism Involved in Its Anti-Atherosclerotic Effect. *Open Cardiovascular Medicine Journal*.

[16] Zhang H., Tsao R. (2016). Dietary Polyphenols, Oxidative Stress and Antioxidant and Anti-Inflammatory Effects. *Current Opinion in Food Science*.

[17] Catarino M. D., Silva A. M. S., Cardoso S. M. (2017). Fucaceae: A Source of Bioactive Phlorotannins. *International Journal of Molecular Sciences*.

[18] Firdaus M., Nurdiani R., Rivai B., Hemassonida W. H., Badzliyah A., Sugiat N. K. (2022). The Glucose Uptake of Type 2 Diabetic Rats by *Sargassum olygocystum* Extract: In Silico and In Vivo Studies. *Journal of Applied Pharmaceutical Science*.

[19] Oh J. H., Kim J., Lee Y. (2016). Anti-Inflammatory and Anti-Diabetic Effects of Brown Seaweeds in High-Fat Diet-Induced Obese Mice. *Nutrition Research and Practice*.

[20] Liu L., Heinrich M., Myers S., Dworjanyn S. A. (2012). Towards a Better Understanding of Medicinal Uses of the Brown Seaweed Sargassum in Traditional Chinese Medicine: A Phytochemical and Pharmacological Review. *Journal of Ethnopharmacology*.

[21] Fernando I. P. S., Jayawardena T. U., Kim H. S. (2019). Beijing Urban Particulate Matter-Induced Injury and Inflammation in Human Lung Epithelial Cells and the Protective Effects of Fucosterol From *Sargassum binderi* (Sonder ex J. Agardh). *Environmental Research*.

[22] Yang E. J., Ham Y. M., Yang K. W., Lee N. H., Hyun C. G. (2013). Sargachromenol From *Sargassum micracanthum* Inhibits the Lipopolysaccharide-Induced Production of Inflammatory Mediators in RAW 264.7 Macrophages. *Scientific World Journal*.

[23] Heo S. J., Jang J., Ye B. R. (2014). Chromene Suppresses the Activation of Inflammatory Mediators in Lipopolysaccharide-Stimulated RAW 264.7 Cells. *Food and Chemical Toxicology*.

[24] Lee J. H., Ko J. Y., Samarakoon K. (2013). Preparative Isolation of Sargachromanol E From *Sargassum siliquastrum* by Centrifugal Partition Chromatography and Its Anti-Inflammatory Activity. *Food and Chemical Toxicology*.

[25] Yoon W. J., Heo S. J., Han S. C. (2012). Anti-Inflammatory Effect of Sargachromanol G Isolated From *Sargassum siliquastrum* in RAW 264.7 Cells. *Archives of Pharmacal Research*.

[26] Heo S. J., Yoon W. J., Kim K. N. (2012). Anti-Inflammatory Effect of Fucoxanthin Derivatives Isolated From *Sargassum siliquastrum* in Lipopolysaccharide-Stimulated RAW 264.7 Macrophage. *Food and Chemical Toxicology*.

[27] Han M., Sun P., Li Y., Wu G., Nie J. (2018). Structural Characterization of a Polysaccharide From *Sargassum henslowianum*, and Its Immunomodulatory Effect on Gastric Cancer Rat. *International Journal of Biological Macromolecules*.

[28] Fernando I. P. S., Jayawardena T. U., Sanjeewa K. K. A., Wang L., Jeon Y. J., Lee W. W. (2018). Anti-Inflammatory Potential of Alginic Acid From *Sargassum horneri* Against Urban Aerosol-Induced Inflammatory Responses in Keratinocytes and Macrophages. *Ecotoxicology and Environmental Safety*.

[29] El-Wakkad A., Hassan N. E. M., Sibaii H., El-Zayat S. R. (2013). Proinflammatory, Anti-Inflammatory Cytokines and Adiponkines in Students With Central Obesity. *Cytokine*.

[30] Sudhakaran M., Doseff A. I. (2020). The Targeted Impact of Flavones on Obesity-Induced Inflammation and the Potential Synergistic Role in Cancer and the Gut Microbiota. *Molecules*.

[31] Wang T., He C. (2018). Pro-Inflammatory Cytokines: The Link Between Obesity and Osteoarthritis. *Cytokine and Growth Factor Reviews*.

[32] Aschoff J. K., Kaufmann S., Kalkan O., Neidhart S., Carle R., Schweiggert R. M. (2015). In Vitro Bioaccessibility of Carotenoids, Flavonoids, and Vitamin C From Differently Processed Oranges and Orange Juices [*Citrus sinensis* (L.) Osbeck]. *Journal of Agricultural and Food Chemistry*.

[33] Ribas-Agustí A., Martín-Belloso O., Soliva-Fortuny R., Elez-Martínez P. (2018). Food Processing Strategies to Enhance Phenolic Compounds Bioaccessibility and Bioavailability in Plant-Based Foods. *Critical Reviews in Food Science and Nutrition*.

[34] Wan L., Jakkilinki P. D., Singer M. R., Bradlee M. L., Moore L. L. (2020). A Longitudinal Study of Fruit Juice Consumption During Preschool Years and Subsequent Diet Quality and BMI. *BMC Nutrition*.

[35] Jin Y., Cui X., Singh U. P. (2010). Systemic Inflammatory Load in Humans Is Suppressed by Consumption of Two Formulations of Dried, Encapsulated Juice Concentrate. *Molecular Nutrition and Food Research*.

[36] Hosseini B., Berthon B. S., Saedisomeolia A. (2018). Effects of Fruit and Vegetable Consumption on Inflammatory Biomarkers and Immune Cell Populations: A Systematic Literature Review and Meta-Analysis. *American Journal of Clinical Nutrition*.

[37] Murwani S., Ali M., Muliartha K. (2013). Diet aterogenik pada tikus putih (*Rattus novergicus* strain Wistar) sebagai model hewan aterosklerosis. *Jurnal Kedokteran Brawijaya*.

[38] Fernández-Macías J. C., Ochoa-Martínez A. C., Varela-Silva J. A., Pérez-Maldonado I. N. (2019). Atherogenic Index of Plasma: Novel Predictive Biomarker for Cardiovascular Illnesses. *Archives of Medical Research*.

[39] Tsikas D. (2017). Assessment of Lipid Peroxidation by Measuring Malondialdehyde (MDA) and Relatives in Biological Samples: Analytical and Biological Challenges. *Analytical Biochemistry*.

[40] Firdaus M., Nurdiani R., Artasasta I. N., Mutoharoh S., Pratiwi O. N. I. (2020). Potency of Three Brown Seaweeds Species as the Inhibitor of RNA-Dependent RNA Polymerase of SARS-CoV-2. *Revista de Chimie*.

[41] Kamanna V. S., Kashyap M. L. (2008). Mechanism of Action of Niacin. *American Journal of Cardiology*.

[42] Kim D., Yan J., Bak J., Park J., Lee H., Kim H. (2022). *Sargassum thunbergii* Extract Attenuates High-Fat Diet-Induced Obesity in Mice by Modulating AMPK Activation and the Gut Microbiota. *Foods*.

[43] Firdaus M., Nurdiani R., Savira A., Hanifah F. (2023). *Sargassum aquifolium* Extract Prevents Elevated Cholesterol Levels and Blood Vessel Thickening in Rats Fed an Atherogenic Diet. *Journal of Pure and Applied Chemistry Research*.

[44] Awang A. N., Ng J. L., Matanjun P., Sulaiman M. R., Tan T. S., Ooi Y. B. H. (2014). Anti-Obesity Property of the Brown Seaweed, *Sargassum polycystum* Using an In Vivo Animal Model. *Journal of Applied Phycology*.

[45] Woo M. N., Jeon S. M., Kim H. J. (2010). Fucoxanthin Supplementation Improves Plasma and Hepatic Lipid Metabolism and Blood Glucose Concentration in High-Fat Fed C57BL/6N Mice. *Chemico-Biological Interactions*.

[46] Wuenstel J. W., Wądołowska L., Słowińska M. A., Niedźwiedzka E., Kowalkowska J., Antoniak L. (2015). Consumption Frequency of Fruit Juices and Sweetened Beverages: Differences Related to Age, Gender and the Prevalence of Overweight Among Polish Adolescents. *Polish Journal of Food and Nutrition Sciences*.

[47] Petric Z., Žuntar I., Putnik P., Bursać Kovačević D. (2021). Food–Drug Interactions With Fruit Juices. *Foods*.

[48] Liu K., Xing A., Chen K. (2013). Effect of Fruit Juice on Cholesterol and Blood Pressure in Adults: A Meta-Analysis of 19 Randomized Controlled Trials. *PLoS One*.

[49] Motshakeri M., Ebrahimi M., Goh Y. M., Matanjun P., Mohamed S. (2013). *Sargassum polycystum* Reduces Hyperglycaemia, Dyslipidaemia and Oxidative Stress Via Increasing Insulin Sensitivity in a Rat Model of Type 2 Diabetes. *Journal of the Science of Food and Agriculture*.

[50] Chen Z., Xu Y., Liu T., Zhang L., Liu H., Guan H. (2016). Comparative Studies on the Characteristic Fatty Acid Profiles of Four Different Chinese Medicinal Sargassum Seaweeds by GC-MS and Chemometrics. *Marine Drugs*.

[51] Gerasimenko N., Logvinov S. (2016). Seasonal Composition of Lipids, Fatty Acids Pigments in the Brown Alga *Sargassum pallidum* The Potential for Health. *Open Journal of Marine Science*.

[52] Adiels M., Chapman M. J., Robillard P. (2018). Niacin Action in the Atherogenic Mixed Dyslipidemia of Metabolic Syndrome: Insights From Metabolic Biomarker Profiling and Network Analysis. *Journal of Clinical Lipidology*.

[53] Lee Y. W., Kim P. H., Lee W. H., Hirani A. A. (2010). Interleukin 4, Oxidative Stress, Vascular Inflammation and Atherosclerosis. *Biomolecules & Therapeutics (Seoul)*.

[54] Zhao T. X., Mallat Z. (2019). Targeting the Immune System in Atherosclerosis: JACC State-of-the-Art Review. *Journal of the American College of Cardiology*.

[55] Saigusa R., Winkels H., Ley K. (2020). T Cell Subsets and Functions in Atherosclerosis. *Nature Reviews Cardiology*.

[56] Yoon J. Y., Kim H. R. (2015). The Immunomodulatory Effects of Brown Seaweed Extract on CD4+ T Cells. *Journal of Ethnopharmacology*.

[57] Park M., Kim J. H., Han J. S. (2018). Anti-Inflammatory Effects of Fucoidan on Hypothalamic Inflammation in High-Fat Diet-Induced Obese Mice. *Journal of Medicinal Food*.

[58] Nascimento D. M., Ferreira A. M., dos Santos C. P., da Costa L. F., Lima T. S., de Almeida F. C. (2019). Fucoidan From Brown Seaweed Enhances IL-4 Production and Promotes Th2 Cell Differentiation. *Food Research International*.

[59] Smith A. B., Lee J., Kim S. H. (2019). Immunomodulatory Effects of *Schisandra chinensis* Extract on CD4+ T cell IL-4 Expression in Hypercholesterolemic Rats. *Journal of Functional Foods*.

[60] Johnson M. C., Nguyen T. H., Parker S. J. (2020). Effects of *Schisandra chinensis* Juice Administration Frequency on Immune Response in Hypercholesterolemic Rats. *Phytotherapy Research*.

[61] Chen L., Liu R., He X., Pei S., Li D. (2021). Effects of Brown Seaweed Polyphenols, a Class of Phlorotannins, on Metabolic Disorders: Via Regulation of Fat Function. *Food and Function*.

[62] Choi E. W., Lee M., Song J. W. (2020). *Fas* Mutation Reduces Obesity by Increasing IL-4 and IL-10 Expression and Promoting White Adipose Tissue Browning. *Scientific Reports*.

[63] Giri S. S., Sen S. S., Sukumaran V., Park S. C. (2018). Niacin Ameliorates Chemically Induced Colitis in Rats by Inhibiting the Production of Proinflammatory Cytokines and Downregulating the Expressions of NF-*κ*B and MAPK Signaling Pathways. *International Immunopharmacology*.

[64] Singh N., Gurav A., Sivaprakasam S. (2019). Activation of the Receptor (Gpr109a) for Niacin and the Commensal Metabolite Butyrate Suppresses Colonic Inflammation and Carcinogenesis. *Immunity*.

[65] Zhao Y., Zhang M. (2020). The role of TNF-*α* in the Differentiation and Activation of CD4+ T Cells Under Hypercholesterolemic Conditions. *Journal of Immunology Research*.

[66] Mauro C., Smith J., Cucchi D. (2017). Obesity-Induced Metabolic Stress Leads to Biased Effector Memory CD4+ T Cell Differentiation via PI3K p110*δ*-Akt-Mediated Signals. *Cell Metabolism*.

[67] Sohrabi Y., Lagache S. M., Schnack L. (2018). Downregulation of CD4+ T Cell Responses by the Cholesterol Biosynthesis Pathway. *Journal of Immunology*.

[68] González-Guerrero A., Gámez-López A. L., Villanueva R. (2019). Differential Expression of TNF-*α* by CD4+ T Cells in Normal and Hypercholesterolemic Mice. *Clinical and Experimental Immunology*.

[69] Jiang Y., Wang Z., Ma B. (2019). High-Fat Diet Induces CD4+ T Cell-Mediated Inflammatory Responses in Obesity. *Journal of Leukocyte Biology*.

[70] Sanjeewa K. K. A., Lee J. S., Kim W. S., Jeon Y. J. (2019). The Potential of Brown-Algae Polysaccharides for the Development of Anti-Cancer Agents: An Update on Anti-Cancer Effects Reported for Fucoidan and Laminaran. *Carbohydrate Polymers*.

[71] Fernando I. P. S., Jayawardena T. U., Sanjeewa K. K. A., Wang L., Jeon Y. J., Lee W. W. (2016). Anti-Inflammatory Potential of Alginic Acid From *Sargassum horneri* Against Urban Aerosol-Induced Inflammatory Responses in Keratinocytes and Macrophages. *Journal of Applied Phycology*.

[72] Lee H. S., Lee S. H. (2015). Fucoidan From Brown Seaweed Induces TNF-*α* Production in Macrophages Through NF-*κ*B Signaling Pathway. *Journal of Medicinal Food*.

[73] Ahn J. H., Kim B. N., Yi S. J. (2019). Protective Effect of *Sargassum serratifolium* Extract on UVB-Induced Inflammation and Oxidative Stress in HaCaT Human Keratinocytes. *Journal of Microbiology and Biotechnology*.

[74] Agarwal P., Alok S., Verma A. (2018). Regulation of Tumor Necrosis Factor-Alpha (TNF-*α*) Expression by Polyphenols: A Review. *Current Pharmaceutical Biotechnology*.

[75] Liu Y., Wang H., Cai L., Ma J., Zeng X. (2019). Schisandrin B Inhibits TNF-*α* Expression Through Suppression of NF-*κ*B and MAPK Signaling Pathways in Lipopolysaccharide-Induced RAW 264.7 Macrophages. *Inflammation*.

[76] Shen P., Zhang Z., He Y. (2018). Magnolol Treatment Attenuates Dextran Sulphate Sodium-Induced Murine Experimental Colitis by Regulating Inflammation and Mucosal Damage. *Life Sciences*.

[77] Kim H. J., Lee Y. S., Park J. H. (2018). Anti-Inflammatory Effects of *Schisandra chinensis* Extract on TNF-*α* Expression in Hypercholesterolemic Mice. *Journal of Medicinal Food*.

[78] Patel S., Rauf A., Khan H., Khalid S., Mubarak M. S. (2020). Potential Health Benefits of Natural Products Derived From *Schisandra chinensis*. *Journal of Food Biochemistry*.

[79] Linke A., Sonnabend M., Fasshauer M. (2017). Effects of Extended-Release Niacin on Lipid Profile and Inflammatory Markers in Patients With Metabolic Syndrome. *Atherosclerosis*.

[80] Drew B. G., Rye K. A. (2014). Niacin Modulates TNF-*α* and CD4+ T Cell Activation in Patients With Dyslipidemia. *Atherosclerosis*.

[81] Navab M., Anantharamaiah G. M. (2018). Niacin's Effect on CD4+ T Cell Activity and TNF-*α* Production in Hyperlipidemic Individuals. *American Journal of Cardiology*.

[82] Chai S. C., Foley E. M., Bortner C. D. (2018). Niacin Suppresses Progression of Atherosclerosis by Inhibiting Vascular Inflammation and Apoptosis of Vascular Smooth Muscle Cells. *Scientific Reports*.

[83] Sharma A. M., Stoll M. (2019). The Role of Niacin in Modulating TNF-*α* Expression by CD4+ T Cells in Hypercholesterolemia. *Journal of Lipid Research*.

[84] Li X., Ballantyne C. M., Bhatnagar S. (2014). The Anti-Inflammatory Effects of Niacin on CD4+ T Cell-Mediated TNF-*α* Production in Hyperlipidemia. *Journal of Clinical Investigation*.

[85] Parodi-Rullán R., Tarragó M. G. (2017). Niacin-Mediated Reduction of TNF-*α* Expression Through GPR109A Signaling in Hypercholesterolemic CD4+ T Cells. *Journal of Lipid Research*.

[86] Gille A., Offermanns S. (2013). Role of Niacin in Regulating TNF-*α* Expression in T Cells Under Hypercholesterolemic Conditions Via GPR109A Receptor. *Molecular Pharmacology*.

[87] Grebe A., Hoss F., Latz E. (2018). NLRP3 Inflammasome and the IL-1 Pathway in Atherosclerosis. *Circulation Research*.

[88] Ferreira N. S., Bruder-Nascimento T., Pereira C. A. (2020). NLRP3 Inflammasome and Mineralocorticoid Receptors are Associated With Vascular Dysfunction in Type 2 Diabetes Mellitus. *Cells*.

[89] Huang W. Y., Yang J. L. (2016). The Anti-Inflammatory Effects of Brown Seaweed Extract on IL-1*β* Production in Macrophages. *Journal of Ethnopharmacology*.

[90] Rohm T. V., Meier D. T., Olefsky J. M., Donath M. Y. (2022). Inflammation in Obesity, Diabetes, and Related Disorders. *Immunity*.

[91] Montserrat-de la Paz S., del Carmen Naranjo M., Lopez S. (2023). Immediate-Release Niacin and a Monounsaturated Fatty Acid-Rich Meal on Postprandial Inflammation and Monocyte Characteristics in Men With Metabolic Syndrome. *Clinical Nutrition*.

[92] Firdaus M., Agustin F. P., Kholifatul A. J., Wiratama N., Sudjatmiko Y. A. (2015). Antiobesity of Fucoxanthin From *Sargassum echinocarpum* by Increasing *β*-Oxidation in Adipocyte. *KnE Life Sciences*.

[93] Wanders D., Graff E. C., White B. D., Judd R. L. (2013). Niacin Increases Adiponectin and Decreases Adipose Tissue Inflammation in High Fat Diet-Fed Mice. *PLoS One*.

[94] Youm Y. H., Nguyen K. Y., Grant R. W. (2013). The Ketone Metabolite *β*-Hydroxybutyrate Blocks NLRP3 Inflammasome-Mediated Inflammatory Disease. *Nature Medicine*.

